# The Functional Role of SEC23 in Vesicle Transportation, Autophagy and Cancer

**DOI:** 10.7150/ijbs.37008

**Published:** 2019-09-07

**Authors:** Jingchen Jing, Bo Wang, Peijun Liu

**Affiliations:** 1 Center for Translational Medicine, The First Affiliated Hospital, Xi'an Jiaotong University; 2 The Key Laboratory for Tumor Precision Medicine of Shaanxi Province, The First Affiliated Hospital, Xi'an Jiaotong University, Xi'an, Shaanxi 710061, China

**Keywords:** SEC23, COPII-vesicle transportation, autophagy, cancer

## Abstract

SEC23, the core component of the coat protein complex II (COPII), functions to transport newly synthesized proteins and lipids from the endoplasmic reticulum (ER) to the Golgi apparatus in cells for secretion. SEC23 protein has two isoforms (SEC23A and SEC23B) and their aberrant expression and mutations were reported to cause human diseases and oncogenesis, whereas SEC23A and SEC23B may have the opposite activity in human cancer, for a reason that remains unclear. This review summarizes recent research in SEC23, COPII-vesicle transportation, autophagy, and cancer.

## Introduction

**Figure 1:**
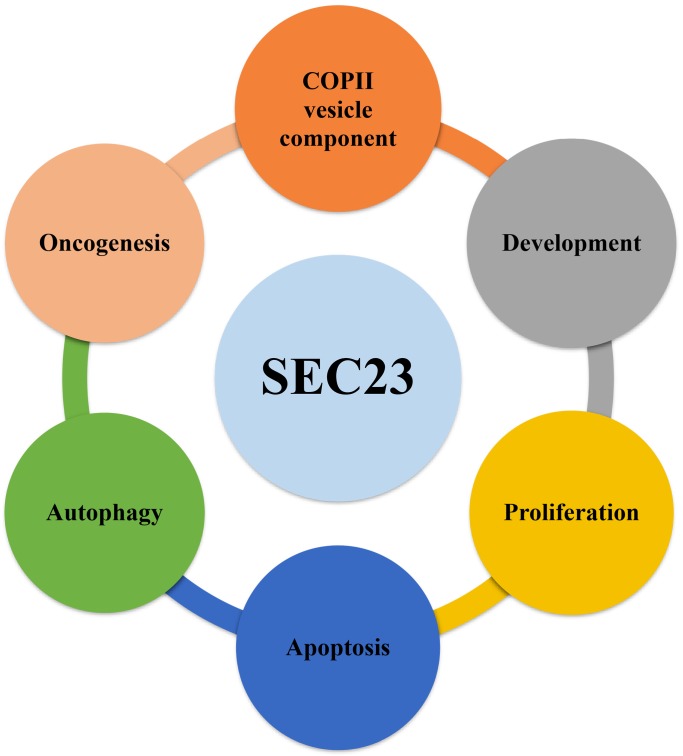
** SEC23 function in cell proliferation, autophagy, and apoptosis.** As a core component of COPII vesicles, SEC23 is not only involved in the protein transportation and secretion process in cells but also participates in autophagy and promotes the survival of cancer cells. Furthermore, SEC23B regulates development of the brain and pancreas in mice.

SEC23 is one of the components in the coat protein complex II (COPII) and regulates the transportation of proteins and lipids from the endoplasmic reticulum (ER) to the Golgi apparatus in cells. Approximately, one-third of all newly synthesized proteins will be transported from the ER to the Golgi through COPII-coated vesicles 1. The SEC23 protein, as the core component of COPII, contains five distinct domains (i.e., the zinc finger, trunk domain, β-barrel, α-helix, and gelsolin domain) and has two isoforms (SEC23A and SEC23B). SEC23 is a GTPase-activating protein (GAP) that stimulates SAR1-GTP hydrolysis in order to facilitate vesicle transportation *in vivo* 2, which activity could be induced by SEC31 3. A previous study demonstrated that a SEC23B mutation causes congenital dyserythropoietic anemia type-II (CDAII) 4, while the SEC23A mutation causes cranio-lenticulo-sutural dysplasia (CLSD) due to abnormal endoplasmic-reticulum-to-Golgi trafficking 5. Aberrant expression was reported to be associated with human cancer development, whereas SEC23A and SEC23B may have the opposite activity in human cancer, for a reason that remains unclear 1. Thus, in this review, we summarized recent research involving SEC23, COPII-vesicle transportation, autophagy, and cancer development and progression (Fig. 1).

### Structure of SEC23

**Figure 2:**
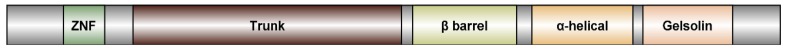
** Illustration of SEC23 protein functional domains.** (Modified from Lee and Miller 10 and Yoshihisa et al. 11, using the tools from a previous study 85). The SEC23 protein contains five functional domains, i.e., the zinc finger, trunk domain, β-barrel, α-helix, and gelsolin-like domain 10, 11.

**Figure 3:**
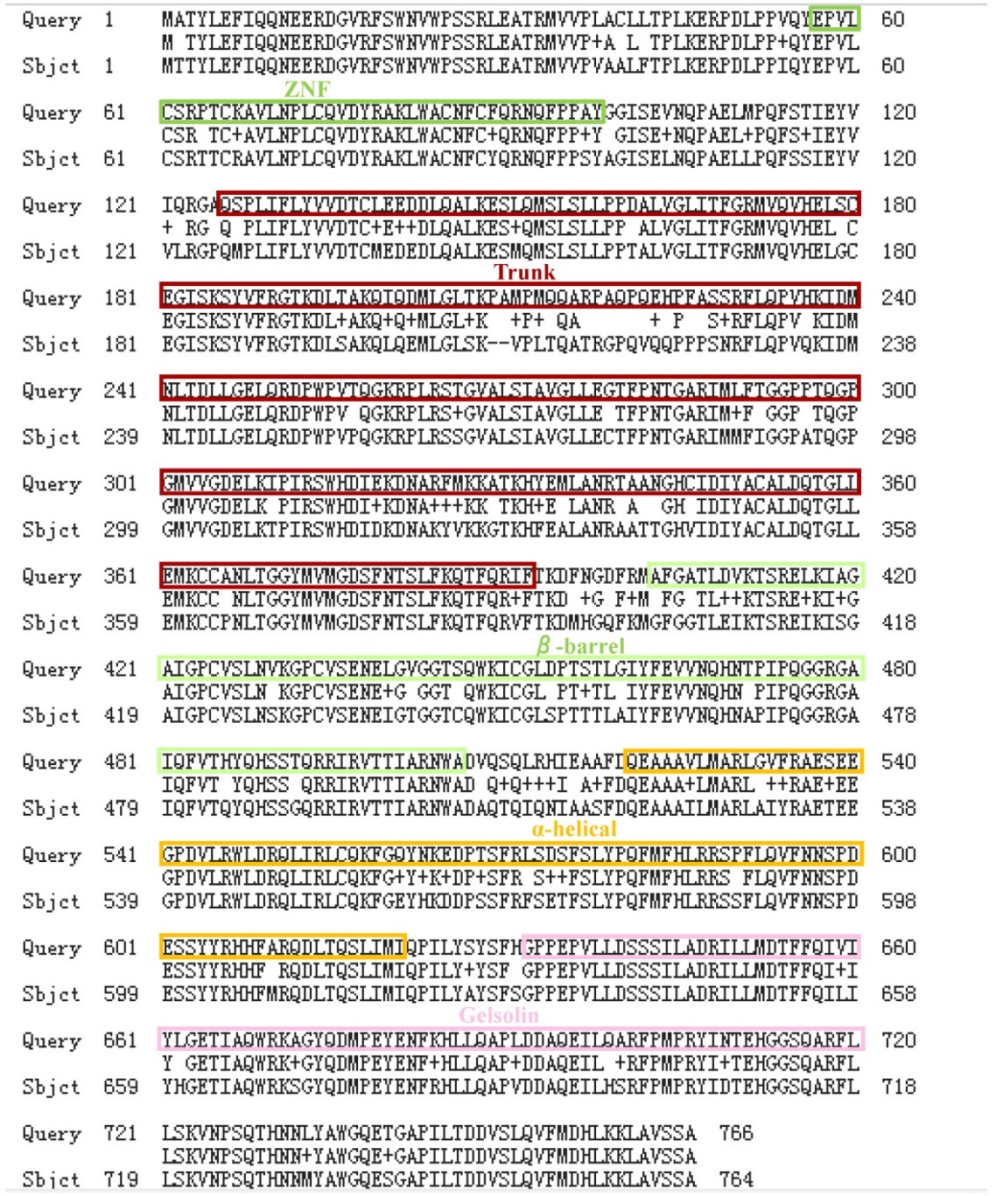
** Amino acid blast results of SEC23A and SEC23B.** The five functional domains of SEC23B 41 are labeled by different colors, while SEC23A could not be labeled for its unknown domain location. Refer to the structure of SEC23 12, the domain location of SEC23A may be similar to SEC23B.

There are two isoforms of SEC23, namely SEC23A and SEC23B, to interact with SEC24 6 and SEC16 7, 8. Both hSEC23A and hSEC23B have a molecular mass of approximately 85 kDa (765 vs. 767 amino acids) with 85% homology 9, exceeding 95% similarities between mouse and human SEC23A and SEC23B. The functions of SEC23A and SEC23B in the COPII complex were interchangeable *in vivo* 1. The human *SEC23A* gene is localized at chromosome 14q21.1 with 22 exons, while *SEC23B* is localized at chromosome 20p11.23 (https://www.genecards.org/). The SEC23 protein contains five distinct domains (i.e., the zinc finger, trunk domain, β-barrel, α-helix, and gelsolin-like domain; Fig. 2) 10, 11. The zinc finger domain has 55 amino acids, containing a series of the zinc fingers that can interact with RNA and DNA, while the trunk domain consists of 250 amino acids and is able to interact with Sar1 and the β-barrel domain consists of 180 amino acids and is essential to form the inner coat. Moreover, the α-helical domain (with 105 amino acids) interacts with Sar1 12 and the 105 amino acid gelsolin-like domain is able to bind to the C-terminus of SEC31, which shares the same triple-proline motifs with SEC16 13, TANGO1, and cTAGE5 14, 15. However, after we searched and blasted the functional domains of SEC23A and SEC23B, we found that there are differences in the regions of their five functional domains, like the Trunk domain, which could lead to some different functions between these two proteins (Fig. 3). In contrast, these two proteins do have many identical regions and in some situations, they may compensate each other in cells. Thus, these two proteins are siblings, they do have differences in functions and expression in tissues; for example, the level of SEC23B protein in the pancreas was higher by approximately nine folds than SEC23A did, whereas SEC23A in the liver was much higher than SEC23B was. Thus, the differential expression of these two isoforms in a given organ or tissue could lead to their distinct phenotype or functions obviously.

## SEC23 Regulation of COPII-vesicle Transportation

In normal eukaryotic cells, the ER functions to facilitate protein synthesis, folding, and dispatch in the rough ER or lipid synthesis in the smooth ER 16. The newly translated proteins destined for secretion are transferred to the Golgi apparatus for post-translational modification and then either transferred to different cell compartments or secreted into the extracellular space 17, while lipids are also transported to other organelles or secreted 18. However, before being transferred to the Golgi, they are selected and packaged into the COPII to make COPII-coated vesicles 19, 20, and the COPII physically deforms the ER membrane into vesicles and selects cargo molecules to transport them to the Golgi. To date, the COPII is shown to consist of five proteins (i.e., SAR1, SEC23, SEC24, SEC13, and SEC31). GTP-loaded SAR1, a small GTPase, will bind to the ER membrane and recruit the SEC23/SEC24 complex to form the inner coat. The SEC23/SEC24 complex will recruit the SEC13/SEC31 complex (the outer coat) for complete assembly of COPII 21. After that, the COPII-containing vesicles leave the ER membrane and SEC23 functions as GAP for SAR1 dissociation from COPII 22, leading to the formation of the ER-Golgi intermediate compartment, along which, the vesicles enter the Golgi apparatus 23. This observation indicates that SEC23 is a core component of COPII and is involved in the formation of the inner coat by acting as a GAP and participating in cargo selection 7.

It is well established that the SEC23/SEC24 complex is recruited to the ER membrane by SAR1, a small G protein, while SEC23A cycling can be altered by ER stress, which will reduce the stability of SEC23 proteins and their binding to the ER membrane 24. After being recruited to the ER membrane, SEC23 will act as a GAP for SAR1, while SEC24 will select and capture most of the cargo 25. Indeed, a previous structural analysis study demonstrated that the size of the COPII vesicles was usually between 60 and 100 nm and that the size of the COPII vesicles bound to the concave surface of SEC23/SEC24 and SAR1 was approximately 60 nm 11. Moreover, SEC23, residing at the inner and outer coats, will interact with SEC31, residing at the outer COPII coat, to form a SEC23-SEC31 interface in order to recognize and capture various cargo molecules 26. Molecularly, the Phe380 residue of the SEC23 protein forms the binding groove to accommodate SEC31 interaction and promotion of SEC23 GAP activity and COPII disassembly 27. In addition, SEC23 can also interact with the p150 dynactin subunit and the dynein motor in order to facilitate microtubule-driven traffic from the ER-Golgi intermediate compartment 28, 29. However, other previous studies showed that Rab1b, localized at the ER-Golgi interface and the Golgi 30, 31, was able to interact with SEC23 and change the kinetics of COPII association/dissociation at the ER exit site 32.

Overall, SEC23 and the COPII-vesicle play an important role in the transportation of newly translated proteins or lipids that are destined for secretion in cells. Indeed, during cancer development and progression, a great number of proteins are produced and transported in cancer cells, and SEC23B may play an important role in this process.

## SEC23 Regulation of Embryo Development

Previous studies demonstrated that SEC23B played an essential role in mouse embryo development, especially in the development of the secretory tissues, like the pancreas 33, 34. SEC23B-knockout mice died shortly after birth, due to the degeneration of the secretory tissues 34. *SEC23B* knockout not only led to an accumulation of proteins in the ER lumen but also activated the apoptosis pathway in response to unfolded proteins (i.e., the ER stress response) 34. The mice with SEC23B deficiency had smaller pancreas than the wild-type mice, and their pancreas structures also showed abnormalities, such as a lack of zymogen granules in exocrine cells 33, 34.

In addition, a previous study showed that MIA SH3 Domain ER Export Factor 2 (MIA2) also participated in brain development 35, 36 and protein secretion in embryos and loss of MIA2 expression affected the interaction between SAR1 and SEC23, leading to the persistent SAR1 activation to interrupt COPII vesicle formation and transportation from the ER to the Golgi in neurons 37. Similarly, knockout of SEC23A expression resulted in death of mice during embryonic stages with defects in embryo and neural tube development 38.

Furthermore, it was reported that SEC23B was able to regulate cell growth. For example, epidermal growth factor (EGF), functioning in cell growth and differentiation, can upregulate SEC23B expression through the transcriptional regulator RNF11 39. EGF treatment was able to induce the transportation efficiency of newly synthesized EGFRs from the ER to the cell membrane by upregulation of SEC23B, SEC24B, and SEC24D 39. This was an important process for maintaining physiological levels of EGFR in cells, otherwise it could lead to the proliferation of human cancer cells. It was also reported that SEC23 could directly interact with the Trk-fused gene (TFG) through the TFG C-terminus in order to induce outer coat dissociation 40. TFG was implicated in multiple neurodegenerative diseases and oncogenesis 40; thus, SEC23 interaction with TFG could lead to tumor cell growth.

Thus, SEC23 could regulate embryo development, especially the secretory tissues and the neural system. It could also promote cancer cell growth and inhibit cell apoptosis, indicating that SEC23 may accelerate cancer development and progression.

## *SEC23* Mutations in Human Diseases

To date, various mutations associated with SEC23A and SEC23B were reported in human diseases 10, although there was no two null *SEC23B* alleles occurring in patients, indicating that complete *SEC23B* deficiency could be lethal for cells 41. Previous studies revealed *SEC23A* mutations in CLSD, which is an autosomal recessive disease 5, 42. CLSD patients have craniofacial and skeletal malformations, associated with defects in collagen secretion 5, 42. The germline *SEC23A* deletion in mice showed the same phenotype of human CLSD 38, while *SEC23A* mutations in zebra fish jeopardized cartilage development 43.

On the other hand, *SEC23B* mutations could cause congenital CDAII in human beings, which is an autosomal recessive disease. This disease is characterized by moderate anemia and a lack of erythropoiesis 4, 44. However, mice with complete SEC23B deficiency did not show visible anemia or any of the characteristics of CDAII 34, although the mice would be unable to survive after birth, probably because of hypoglycemia 34. In contrast, insertion of the *SEC23A* coding sequences into the murine *SEC23B* locus was able to rescue the lethal SEC23B-deficient pancreatic phenotype completely in mice 1. Furthermore, an increase in SEC23A expression could also compensate for the function of mutated SEC23B in CDAII patients, a potential therapeutic strategy for CDAII patients 45, 46. In addition, *SEC23B* mutations showed completely different phenotypes among humans, mice, and zebra fish 1. In human tissues, SEC23B was mainly expressed in the bone marrow, whereas SEC23B was strongly expressed in the mouse pancreas 13, 14 and disruption of SEC23B expression showed defects in the secretion of extracellular matrix protein in zebra fish, which mimics human CLSD 4.

Furthermore, approximately 3-9% of all thyroid cancer cases are a familial non-medullary thyroid cancer (FNMTC), which may be due to Cowden syndrome 47, a rare autosomal dominant familial cancer syndrome with a high risk of developing breast cancer, metrocarcinoma, and non-medullary thyroid cancer 48. A previous study of the whole exome sequences in FNMTC revealed that SEC23B was a novel susceptibility gene in Cowden syndrome and identified a heterozygous missense SEC23B variant (c. 1781T>G [p.Val594Gly]) in a multi-generation Cowden syndrome family 49.

Therefore, both SEC23A and SEC23B have vital functions in human beings, loss of which would cause serious diseases; particularly, lost SEC23B closely associated with Cowden syndrome, which has potentially cancer predisposing.

## SEC23 Regulation of Autophagy in Cells

The transport protein particle (TRAPP) III complex could be recruited to the phagophore assembly site of COPII during macroautophagy and bind to SEC23, providing the membrane components to form the autophagosome and autophagy 50. Moreover, SEC23 protein could be phosphorylated by a serine/threonine protein kinase Hrr25 to trigger autophagy-related pathways; however, if there is a loss of SEC23 expression, starvation-induced autophagy could be impaired 51. A previous study has demonstrated that autophagy was controlled by a novel mechanism based on ULK-FBXW5-SEC23B action, showing that F-Box and WD Repeat Domain Containing 5 (FBXW5) could inhibit biogenesis of the COPII-mediated autophagosome by targeting and promoting SEC23B degradation in the presence of nutrients, whereas ULK1 phosphorylated SEC23B at S186 and prevented SEC23B and FBXW5 interaction in order to inhibit SEC23B degradation during cell starvation, which in turn led to cell autophagy 52. The UNC51-like kinase 1 (ULK1) is an important enzyme in the regulation of autophagy in mammalian cells 53, 54 and is activated after nutrient deprivation through several upstream signals to initiate autophagy processes 55. Interestingly, a mutation at the SEC23B S186 site, a cancer-related mutation occurring in human melanoma, could abolish the interaction between SEC23B and FBXW5, resulting in an increase in autophagy and promoting the survival of cancer cells 52.

Similarly, ULK1 can phosphorylate of SEC23A to decrease the interaction between SEC23A and SEC31A and suppress the traffic from the ER to Golgi apparatus. Again, ULK1 can also phosphorylate SEC23A at the residues serine 207 and threonine 405 to induce cell autophagy 56. Interestingly, SEC23A and SEC23B are associated with autophagy, which is involved in cancer development and progression.

## SEC23 Alterations in Human Cancers

Thus far, we discussed the function of SEC23 in cells and its alterations and associated mutations in human disease. This section will discuss the role of SEC23 in human cancer development and progression, although previous studies revealed that SEC23A and SEC23B might have been opposing roles in cancer. In Table 1, we summarized the known miRNAs that may directly affect SEC23 expression.

### SEC23A Alterations in Human Cancer

To date, there is no report in PubMed showing SEC23A alterations in human cancer, but the role of SEC23A in human cancer has been reported as a target of other genes, like miRNAs (see below for details); thus, it remains to be determined how SEC23A regulates the development of human cancer.

miRNAs are a class of small non-coding RNA molecules up to 24 nucleotides in length and function to regulate cell proliferation, differentiation, apoptosis, and tumor metastasis by downregulation of protein-coding gene expression 57. A previous study reported that SEC23A was the target gene of miR-200b, miR-200c, or miR-200s, miR-21, miR-375 58-62. For example, miR-200 played an important role in the regulation of embryonic and cancer stem cells and sensitivity of cancer cells to chemotherapy 63-67. SEC23A is one of the miR-200b targeting genes, which was inversely associated with prostate cancer tissues 59. Moreover, when miR-200c and miR-200s were overexpressed, SEC23A expression was reduced 58, 60. In addition, a recent study revealed that miR-21 expression enhanced colorectal cancer cell proliferation and metastasis by targeting and inhibiting SEC23A expression 68. Furthermore, miR-375 possesses tumor-suppressive activity in different human cancers 69; for example, miR-375 was downregulated in invasive breast cancer, leading to tumor cell migration and invasion 58, 70. Another previous study reported that miR-375 could target the expression of the SEC23A protein by binding to the 3'-untranslatable region (3'-UTR) of *SEC23A* cDNA, while SEC23A expression was reduced in primary prostate carcinoma tissues 61. Furthermore, RNA binding motif 5 (RBM5) functions to regulate gene transcription and mRNA splicing in cells and after knockdown of RBM5 expression in neurons, the level of SEC23A mRNA was upregulated, although there was no significant change in the level of the SEC23A protein 71.

Knockdown of SEC23A expression enhanced tumor cell proliferation and metastatic colonization in prostate and colorectal cancer 58, 59, 61, 68. It was possibly because SEC23A could mediate secretion of Insulin-Like Growth Factor Binding Protein 4 (IGFBP4), a metastasis-suppressive protein 58. Interestingly, SEC23A has a clear inhibitory role in breast cancer metastasis, especially the step of colonization during tumor cell metastasis but not at the step of tumor cell migration. Knockout of SEC23A could increase breast cancer colonization and reduce tumor cell migration, where these two effects of SEC23A may cancel each other out. In fact, endogenous SEC23A was lower in highly metastatic breast cancer cells than in lowly metastatic cells. Consistently, SEC23A was also expressed at a lower level in metastatic than in the primary tumors 60. Taken together, both clinical and animal data demonstrates that SEC23A plays a key role in the inhibition of breast cancer metastasis.

Furthermore, detection of miRNA-375 vs. SEC23A expression in thyroid carcinoma cells was a useful biomarker to assess the *in vitro* efficacy of vandetanib, a selective kinase inhibitor of the vascular endothelial growth factor receptor (VEGFR), the epidermal growth factor receptor (EGFR), and the RET-tyrosine kinase 72. SEC23A expression was also reported to be associated with the resistance of prostate cancer to docetaxel treatment 73. In addition, SEC23A was used as a biomarker of pain flare in patients with painful bone metastases 74.

### SEC23B Alterations in Human Cancer

Previous studies revealed that germline heterozygous SEC23B variants were potentially cancer predisposing, even though the mutant SEC23B (associated with ER stress-mediated tumorigenesis) did not cause an overall decrease in SEC23B expression 49, 75. Indeed, ER stress is a hallmark of cancer 76, 77. Other previous studies showed that alteration of SEC23B was associated with the development of thyroid cancer 78, hepatocellular cancer 79, and prostate cancer 80.

As a novel susceptibility gene in Cowden syndrome, the SEC23B variant (c. 1781T>G [p.Val594Gly]) was able to promote proliferation, colony formation, survival, and invasion of normal thyroid cells, which was associated with cancer predisposition induced by ER stress 49. Furthermore, another study demonstrated that SEC23B contributed to cancer predisposition through the ribosome biogenesis pathway, independent of SEC23B-mediated COPII functions 75. In addition, tandem mass spectrometry 79 compared the transitional endoplasmic reticulum (tER) in the liver tumor nodules vs. the control rat liver and showed that SEC23B expression was upregulated in tumor ER, and this differential SEC23B expression suggests that SEC23B could be used as a potential novel tumor marker for hepatocellular carcinoma 79.

**Figure 4:**
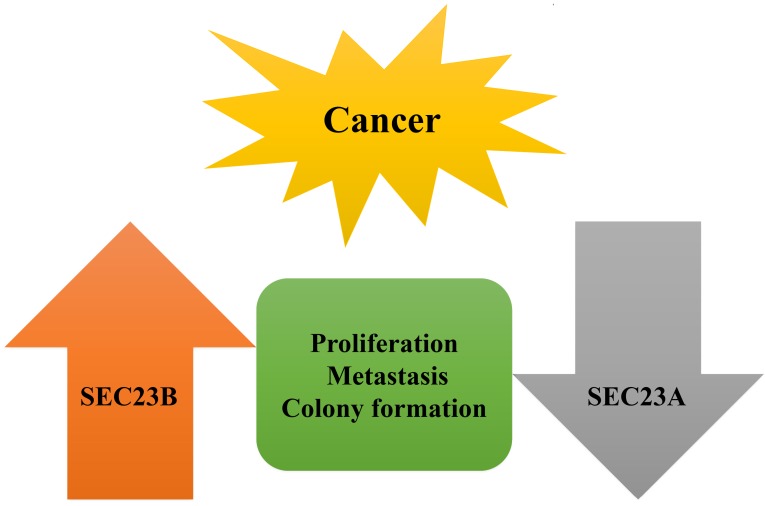
** Opposite functions of SEC23A and SEC23B in human cancer.** SEC23A was expressed at a lower level in cancer tissues and cells, whereas SEC23B expression was often higher in tumor than in normal tissues, indicating their opposite functions in cancer cells, which effects were confirmed in tumor cell proliferation, metastasis, and colony formation.

In addition, SEC23B is shown to be a target of miR-130a and the latter was downregulated in prostate cancer 80, whereas miR-130a overexpression in prostate cancer cells reduced tumor cell proliferation and invasion capacity but induced apoptosis 80. However, knockdown of SEC23B expression mimicked the effect of miR-130a overexpression in prostate cancer cells 80. This study implies that SEC23B could be an oncogene in prostate cancer. Based on these few studies, we concluded that SEC23B expression is frequently upregulated in these types of human cancer and functions as an oncogene or possesses oncogenic activity in these cancers. However, more studies are needed to clarify the role of SEC23B in human cancer (Fig. 4).

## Summary and Future Direction of Research on SEC23

To date, different studies have shown the importance of SEC23 participation and function in COPII, particularly the SEC23/SEC24 complex, in order to facilitate newly translated secretion proteins transferred from the ER to the Golgi apparatus for secretion. SEC23 interacts with SAR1 and SEC31 to select cargo and induce the GTPase activity of SAR1 during vesicle formation, leading to the secretion of proteins and lipids. Thus, as an important member of COPII, SEC23B is involved in collagen secretion 81. SEC23 is involved in embryo development and SEC23B plays an essential role in mouse secretory tissue development, whereas *SEC23B* mutations could lead to the death of the mice shortly after birth. The main reason for this could be that the loss of SEC23 expression enhanced apoptosis of the secretory tissues, like the pancreas, while SEC23 also plays a role in brain development and function in mice. In addition, SEC23 affects on the transportation of EGFR from the ER to the Golgi for cell proliferation.

Interestingly, the loss of SEC23B mainly affects the exocrine glands, not the endocrine tissues. The SEC23B-deficient mice also shows apoptosis in salivary gland and gastric glands, which may be caused by ER stress pathways, such as PERK or other two ways. The loss of SEC23A could also cause the death of mice embryos. And about half of the live SEC23A-deficient mice embryos would appear neural tube opening at midbrain, which could be found in all of the surviving and dead embryos. Besides, the deletion of SEC23A could lead to the apoptosis in extraembryonic membranes, which may be also caused by ER stress, just like SEC23B-deficient mice. Therefore, both SEC23A and SEC23B may have effect on ER stress, especially the PERK pathway.

Furthermore, SEC23B can also help in the regulation of autophagy in cells because SEC23B can be phosphorylated by ULK1, Hrr25, and TRAPPIII, all of which are in the autophagy pathway. ULK1 can also phosphorylate of SEC23A and inhibit the traffic from ER to Golgi. SEC23 was shown to be a master regulator of budding and fusion through the phosphorylation-dephosphorylation cycle in order to facilitate the formation of the autophagosome [f]. Autophagy is an evolutionarily conserved cellular process and its alteration is involved in cancer development and progression 83, 84. *In vitro* SEC23A expression suppressed proliferation and metastasis of breast, prostate, and colorectal cancer cells. In contrast, downregulated SEC23A expression was associated with poor disease relapse-free survival (RFS) of breast cancer patients, indicating that SEC23A might be a potential tumor metastasis suppressor. In addition, reduced SEC23A expression increased sensitivity of medullary thyroid carcinoma to vandetanib treatment, all of which suggest that SEC23A could be a tumor suppressor gene, indicating opposing roles for SEC23A and SEC23B in cancer, although their role in cells is interchangeable *in vivo*.

However, additional research is needed to understand the precise function and role of SEC23A and SEC23B in human disease and cancer development. Future study will analyze their expression using online databases, like TCGA data, to find an association between expression and different human cancers. Functionally, further study will investigate the gain and loss of these proteins' function in cells in order to better understand their role in individual human cancers. Human cell lines remain one of the better models to study and understand SEC23 activity in the context of human disease, while animal experiments could help us to assess the functional role of SEC23.

## Figures and Tables

**Table 1:** SEC23 as the target gene of miRNAs

miR	Target	Cancer type	Ref.
miR-200b	SEC23A	Prostate	58
miR-200c	SEC23A	Prostate	57
miR-200s	SEC23A	Breast	59
miR-21	SEC23A	Colorectal	67
miR-375	SEC23A	BreastProstate	57,60
miR-130a	SEC23B	Prostate	79
